# Identification of SLC40A1, LCN2, CREB5, and SLC7A11 as ferroptosis-related biomarkers in alopecia areata through machine learning

**DOI:** 10.1038/s41598-024-54278-4

**Published:** 2024-02-15

**Authors:** Wen Xu, Dongfan Wei, Xiuzu Song

**Affiliations:** 1 School of Medicine, Zhejiang University, Hangzhou, 310009 China; 2 Department of Dermatology, Hangzhou Third People’s Hospital, Affiliated Hangzhou Dermatology Hospital, Zhejiang University School of Medicine, West Lake Ave 38, Hangzhou, 310009 China

**Keywords:** Alopecia areata, Ferroptosis, Machine learning, Biomarkers, Immune cell infiltration, Computational biology and bioinformatics, Data mining

## Abstract

Alopecia areata (AA) is a common non-scarring hair loss condition driven by the collapse of immune privilege and oxidative stress. The role of ferroptosis, a type of cell death linked to oxidative stress, in AA is yet to be explored, even though it's implicated in various diseases. Using transcriptome data from AA patients and controls from datasets GSE68801 and GSE80342, we aimed to identify AA diagnostic marker genes linked to ferroptosis. We employed Single-sample gene set enrichment analysis (ssGSEA) for immune cell infiltration evaluation. Correlations between ferroptosis-related differentially expressed genes (FRDEGs) and immune cells/functions were identified using Spearman analysis. Feature selection was done through Support vector machine-recursive feature elimination (SVM-RFE) and LASSO regression models. Validation was performed using the GSE80342 dataset, followed by hierarchical internal validation. We also constructed a nomogram to assess the predictive ability of FRDEGs in AA. Furthermore, the expression and distribution of these molecules were confirmed through immunofluorescence. Four genes, namely SLC40A1, LCN2, CREB5, and SLC7A11, were identified as markers for AA. A prediction model based on these genes showed high accuracy (AUC = 0.9052). Immunofluorescence revealed reduced expression of these molecules in AA patients compared to normal controls (NC), with SLC40A1 and CREB5 showing significant differences. Notably, they were primarily localized to the outer root sheath and in proximity to the sebaceous glands. Our study identified several ferroptosis-related genes associated with AA. These findings, emerging from the integration of immune cell infiltration analysis and machine learning, contribute to the evolving understanding of diagnostic and therapeutic strategies in AA. Importantly, this research lays a solid foundation for subsequent studies exploring the intricate relationship between AA and ferroptosis.

## Introduction

Alopecia areata (AA) is a common non-scarring hair loss disorder arising from the collapse of immune privilege^1^. It manifests as rapid hair loss, producing distinct spots of different sizes. Depending on its severity, it's classified into patchy AA (PAA) or non-severe AA (NSAA), and the more intense forms, alopecia totalis (AT) and alopecia universalis (AU), together termed as severe AA (SAA)^2^. Beyond its apparent cosmetic impact, AA is associated with increased risks of mental health challenges and cardiovascular diseases^3,4^.

Recent advances in AA treatment, particularly the development and application of JAK inhibitors, have marked a significant progress in the management of this condition^5,6^. These inhibitors have been instrumental in addressing key aspects of AA's pathogenesis, offering new hope for patients. However, it is noteworthy that some patients experience relapse after discontinuation of these medications, highlighting the importance of understanding AA's underlying mechanisms and the need for early detection^7^. This insight underscores the critical role of continued research in unraveling the complex etiology of AA and enhancing early diagnostic capabilities.

A critical facet of its pathology is the role of oxidative stress, intensifying the disease progression^8^. The role of oxidative stress suggests potential biomarkers for AA diagnosis and treatment. Yet, established clinical markers for AA remain elusive, emphasizing the need for further research in diagnostic and therapeutic modalities.

Although oxidative stress has been recognized in AA, its direct relationship with ferroptosis, a unique form of cell death characterized by lipid peroxidation and iron accumulation^9,10^, has not been definitively established in current literature. Recent studies, such as the work on *Schizochytrium* sp. extracted lipids, illustrate how enhancing antioxidation and inhibiting ferroptosis in dermal papilla cells can be an effective strategy against alopecia^11^. This finding further underscores the potential interplay between oxidative stress and ferroptosis in AA. Additionally , the mechanisms underlying ferroptosis, as detailed by Tang D et al., who identified small molecular compounds such as GPX4, ceruloplasmin, NRF2, and HO-1 that regulate this process^12^, might offer a compelling avenue of investigation for AA. It's well-established that ferroptosis plays a significant role in various diseases, from tumors^13^ and neurodegenerative disorders^14^ to organ damage^15^.

Research directly linking ferroptosis to AA may still be in its infancy. However, the known involvement of oxidative stress—a core component of ferroptosis—in AA provides a compelling foundation. Key studies have shed light on this potential connection. For instance, Sengupta A et al. documented dysmorphic hair follicles and alopecia in perinatal GPx4 knockout mice, a crucial player in ferroptosis^16^. Similarly, Folgueras AR et al. noted distinct hair loss in Tmprss6(-/-) mice, which regulate iron homeostasis, another vital aspect of ferroptosis^17^. Furthermore, evidence from AA patients, such as elevated malondialdehyde and ceruloplasmin levels reported by Cwynar A et al.^18^, and promising results from NRF2 activators in female pattern alopecia observed by Bakry OA et al.^19^ , underscores the relevance of ferroptosis-related markers in the context of AA. Therefore, understanding the role of FRGs in AA may contribute to the development of new diagnostic and therapeutic strategies.

In the age of big data, bioinformatics and machine learning have become cornerstone tools in molecular medical research. Capitalizing on these tools, our study sought to identify marker genes in AA related to ferroptosis. We started by analyzing two GEO datasets: GSE68801^20^ as the training set and GSE80342^21^ as the validation set. The goal was to determine ferroptosis-related differentially expressed genes (FRDEGs) between AA lesions and standard scalp samples. Delving deeper, we explored the relationship between these FRDEGs and immunity, specifically through immune cell infiltration. Employing machine learning techniques, internal hierarchical validation, and external validation, we pinpointed key marker genes. Building on these insights, we constructed an AA prediction model, delved into potential pathways, and forecasted potential effective treatments. Collectively, our research unveils a groundbreaking perspective on AA diagnosis and therapy.

## Materials and methods

**Figure 1:**
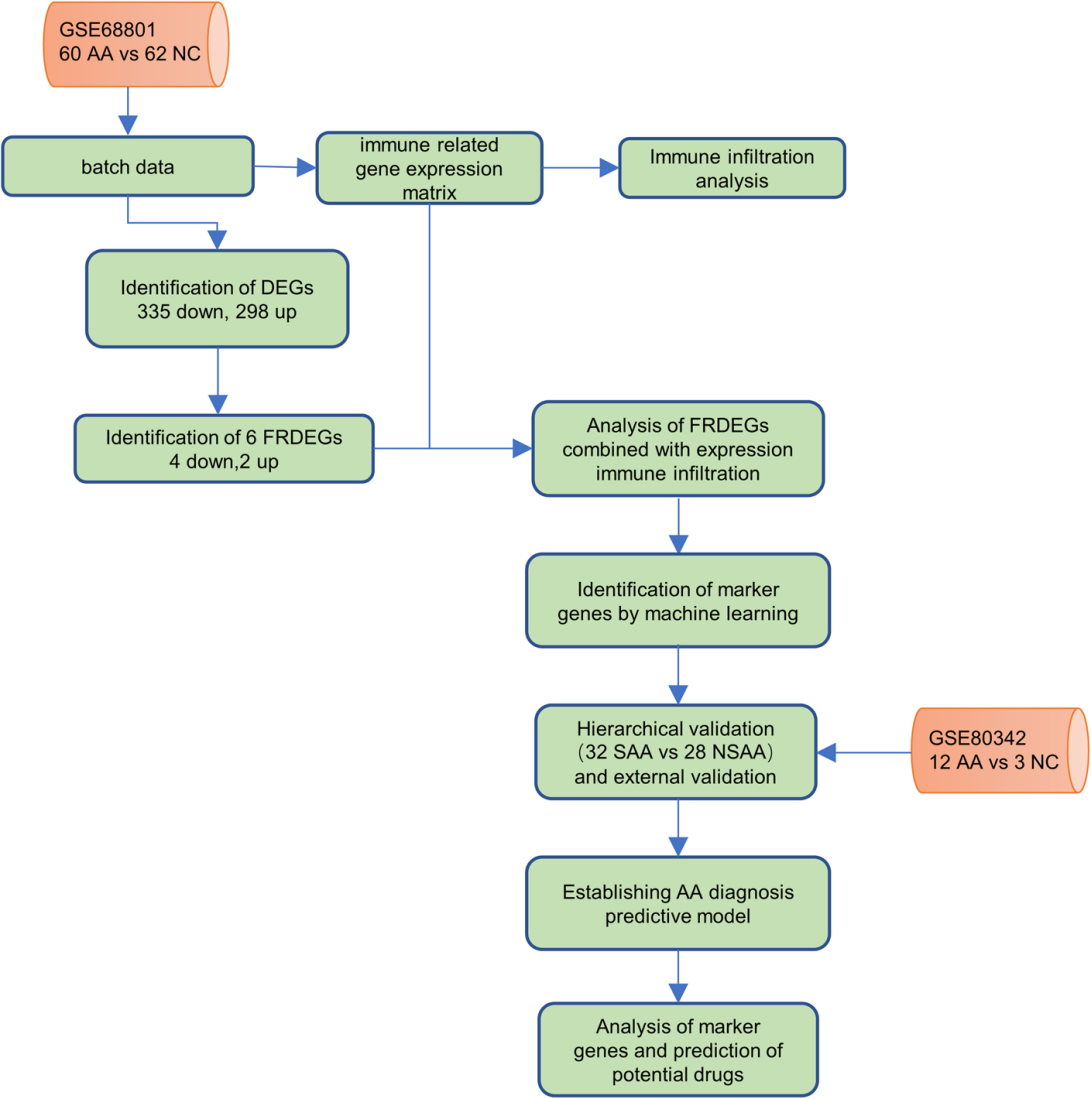
Study Workflow for Analyzing Alopecia Areata (AA). Two datasets were used: GSE68801 (122 samples: 60 AA, 62 NC) as the primary dataset for analysis and GSE80342 (15 samples: 12 AA, 3 NC) for external validation. The workflow involved analyzing differentially expressed genes (DEGs) and ferroptosis-related DEGs (FRDEGs) in these datasets. AA, alopecia areata patients; NC, normal controls; DEGs, differentially expressed genes; FRDEGs, ferroptosis-related differentially expressed genes.

The study's flowchart is illustrated in Fig. 1.

### Data acquisition and processing

Two AA datasets (GSE68801 and GSE80342) were obtained from the GEO database (https://www.ncbi.nlm.nih.gov/geo/). The GSE68801 dataset included 122 samples (60 AA lesions and 62 normal controls (NC)), while GSE80342 contained 15 samples (12 AA lesions and 3 NC). Expression matrix identifiers were converted from 'probe id' to 'symbol' using platform annotation files. Whenever multiple probes matched a single gene symbol, their mean value was computed for expression levels. To correct batch effects, we employed the combat function from the R "SVA" package. While GSE68801 was the main dataset for analysis, GSE80342 was reserved for external validation.

### Accumulation of FRGs

We sourced ferroptosis driver and suppressor genes from FerrDb, the premier database dedicated to ferroptosis regulators and related disease associations (http://www.zhounan.org/ferrdb/current/, accessed on November 21, 2022)^22^. It listed 264 driver genes and 238 suppressor genes, as detailed in Tab. s1 and s2.

### Identification of FRDEGs

Using the "Limma" R package, we analyzed differentially expressed genes (DEGs) between AA lesions and NC scalps in the GSE68801 dataset. DEGs were shortlisted based on criteria: adjusted *p*-value < 0.05 and |log2 fold change|> 0.585^23^.

### ssGSEA analysis

The "gsva" R package facilitated the computation of infiltration scores for 16 immune cells (imc) and 13 immune functions (imf) using ssGSEA, a widely-adopted method for immune cell infiltration^24^. Furthermore, we evaluated associations between FRGs and immune cell infiltration.

### Identification of candidate marker genes

We applied two machine learning techniques to discern potential AA marker genes. Firstly, using the "glmnet" R package, we conducted LASSO regression analysis with tenfold cross-validation, emphasizing genes crucial for distinguishing AA from NC^25^. Secondly, the Support Vector Machine (SVM), a supervised machine learning model, was employed for analyses. To mitigate overfitting risks, recursive feature elimination (RFE) optimized the gene set selection, culminating in SVM-RFE.

### Nomogram construction

Utilizing the R "rms" package, we devised a nomogram by integrating marker genes. Calibration curves assessed its accuracy.

### Immunofluorescence

Scalp tissues from both AA patients and healthy controls were fixed overnight at 4 °C in 4% PFA, subsequently embedded in paraffin, and sectioned. For the preparation of hair follicle sections, we consistently adopted an oblique sectioning angle of 45 degrees relative to the plane of the scalp. This approach was employed to maximize the visibility of hair follicles, allowing for an enhanced assessment of both the follicles and their surrounding as well as intrinsic parameters. Sections were blocked with bovine serum albumin and incubated overnight at 4 °C with antibodies against SLC40A1 (26601-1-AP, Proteintech), LCN2 (26991-1-AP, Proteintech), CREB5 (14196-1-AP, Proteintech), and SLC7A11 (26864-1-AP, Proteintech). After primary antibody exposure, the samples were treated with Alexa Fluor 488-conjugated anti-rabbit IgG (Invitrogen) for 1 h at room temperature, followed by nuclear counterstaining with 4',6-diamidino-2-phenylindole (DAPI; Beyotime). Specimens were visualized and captured using a fluorescence microscope (Olympus).

To quantify the relative fluorescence area (%), the captured images were analyzed using ImageJ software (NIH). The total area of fluorescence was measured for each marker, and this value was expressed as a percentage of the total area of the tissue section under observation. This approach allowed for the standardization of fluorescence intensity measurements across different samples.

To ensure the integrity and stability of the fluorescent signals, experimental procedures involving the handling and processing of fluorescently labeled samples were meticulously conducted in a darkroom setting. Prior to tissue collection, informed consent was obtained from all participants. This study was approved by the Medical Ethics Committee of the Third People's Hospital of Hangzhou (Approval No.: 2022KA058). Both patients and healthy controls were aged 40–60 years. Patients exhibited hair loss covering 40–60% of the scalp area, and none had undergone any treatments in the preceding 6 months.

### Ethics approval and consent to participate

The study was conducted in accordance with the ethical standards of the Declaration of Helsinki and its later amendments. It was approved by the Medical Ethics Committee of the Third People's Hospital of Hangzhou (Approval No.: 2022KA058). Informed consent was obtained from all individual participants included in the study. All methods were carried out in accordance with relevant guidelines and regulations.

### Statistical analysis

In our differential gene analysis, we applied False Discovery Rate (FDR) correction to adjust *p*-values for multiple testing. This ensures the reported adjusted *p*-values minimize the likelihood of false positives, enhancing the reliability of our identified DEGs. For external validation in the GSE80342 dataset, we used Student's t-tests to compare gene expression between AA and NC. Spearman correlation analysis determined correlation coefficients. The R "pROC" package rendered ROC curves and AUC values, gauging the classification efficacy of our predictive model for AA versus NC. All statistical computations were conducted using R software 4.1.1 and Prism (GraphPad Prism, USA). Statistical significance was denoted as *p < 0.05, **p < 0.01, ***p < 0.001, or ****p < 0.0001.

## Results

### Identification of DEGs and FRDEGs

**Figure 2:**
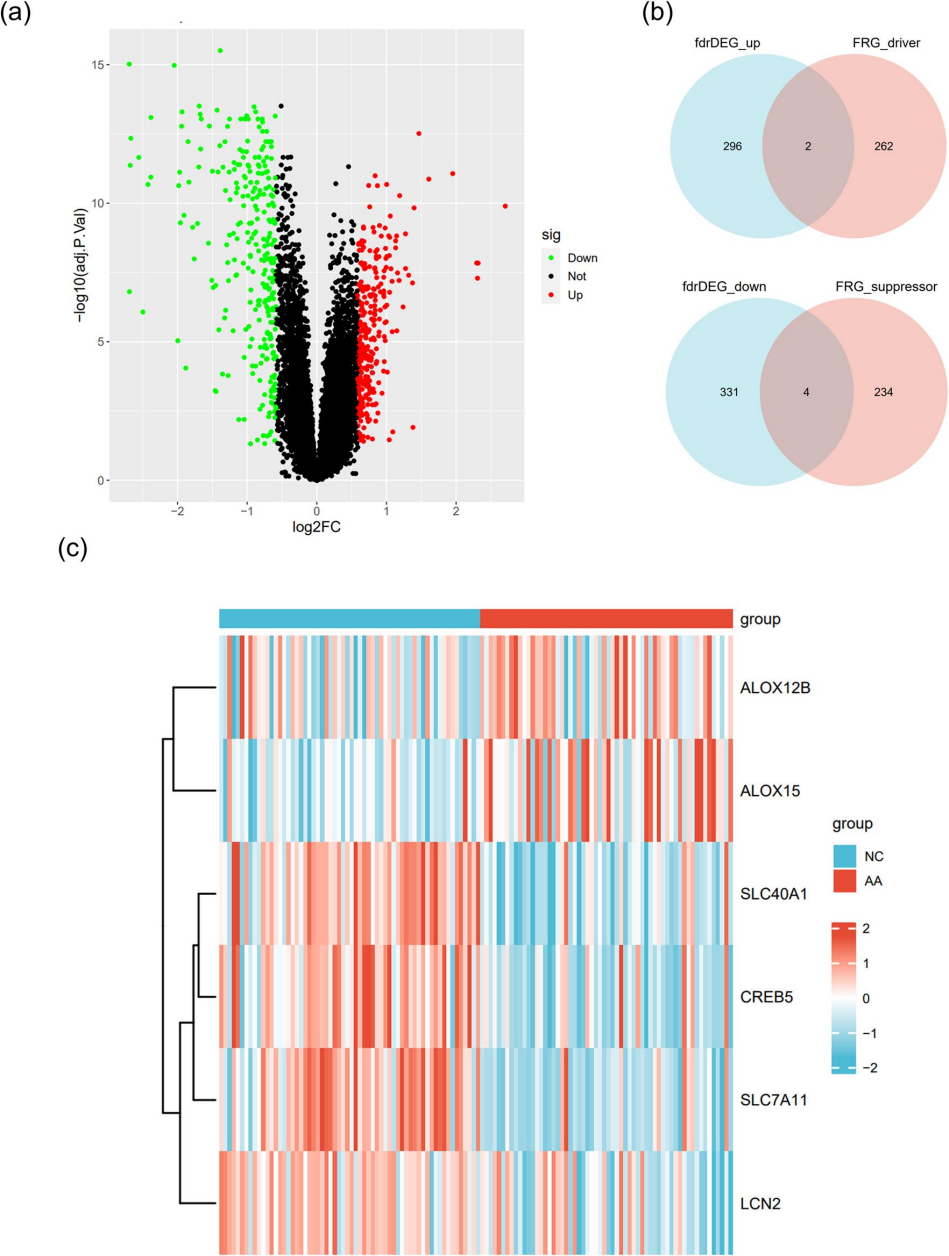
Identification of Ferroptosis-Related Differentially Expressed Genes (FRDEGs) in Alopecia Areata (AA). (**a**) Volcano plot illustrating differentially expressed genes (DEGs) in the GSE68801 dataset, revealing a total of 711 DEGs between AA patients and normal controls (NC), with 355 genes down-regulated and 356 genes up-regulated. (**b**) Venn diagram depicting the overlap between DEGs and ferroptosis-related genes (FRGs), identifying 6 FRDEGs, of which 4 were down-regulated and 2 were up-regulated. (**c**) Heatmap displaying the expression profiles of FRDEGs, highlighting that ALOX15 and ALOX12B are highly expressed in AA, while LCN2, CREB5, SLC7A11, and SLC40A1 are less expressed in AA. The heatmap was generated using R software (version 4.1.1, https://www.r-project.org/). Adjustments to font type and size were made for enhanced clarity. AA, alopecia areata; DEGs, differentially expressed genes; FRGs, ferroptosis-related genes; FRDEGs, ferroptosis-related differentially expressed genes.

A volcano plot (Fig. 2a) revealed 633 DEGs between AA and NC in GSE68801, with 335 down-regulated (green dots) and 298 up-regulated (red dots), as detailed in Tab. s3. A Venn diagram (Fig. 2b) comparing FRGs to DEGs in GSE68801 identified 6 overlapping FRDEGs for further analysis, with 4 down-regulated and 2 up-regulated. A hierarchically clustered heatmap (Fig. 2c) depicted the matrix of FRDEGs in GSE68801.

### Immune cell infiltration analysis in AA

**Figure 3:**
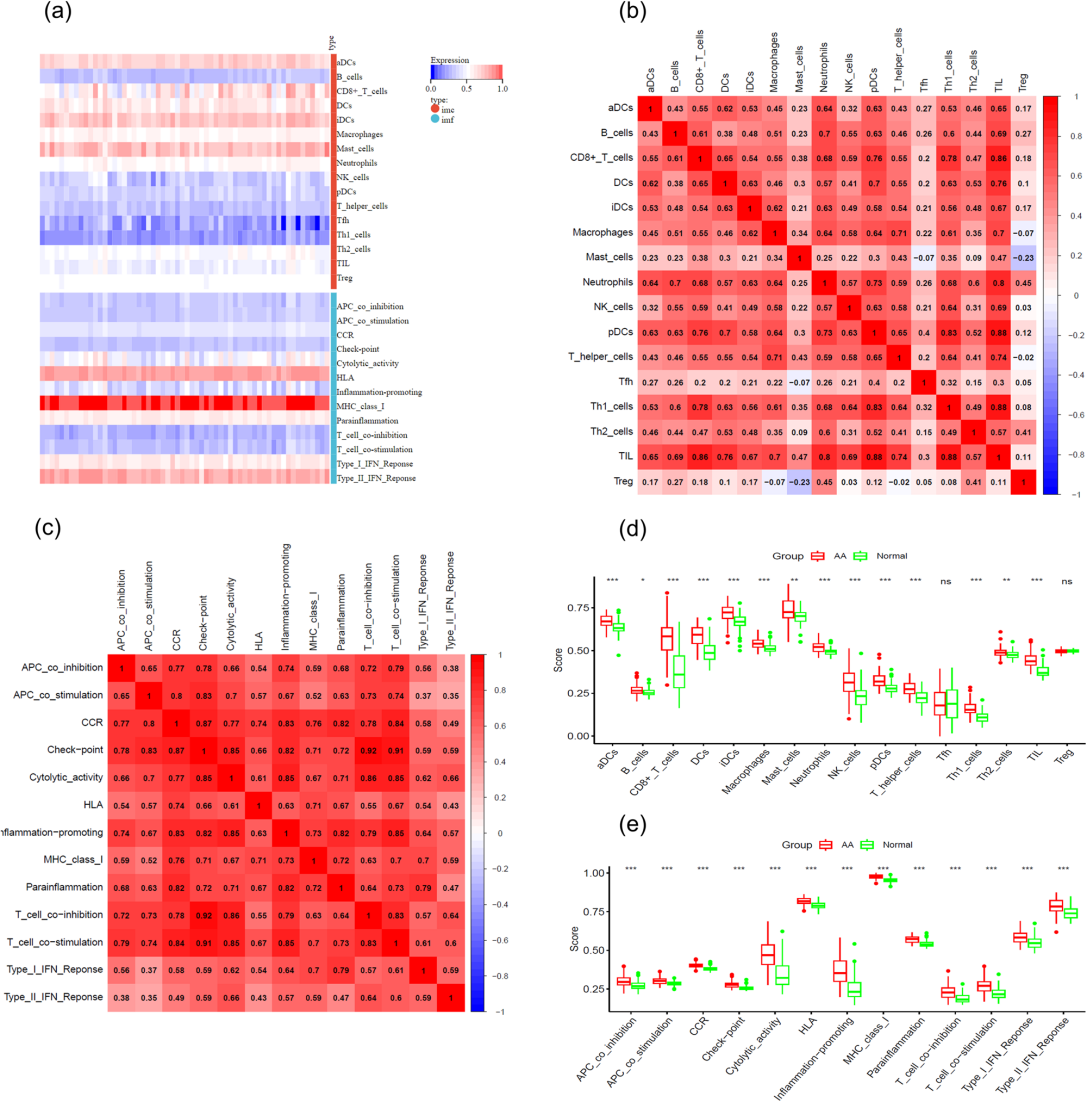
Immune cell infiltration Analysis in Alopecia Areata (AA). (**a**) Heatmap shows 16 immune cell types and 13 immune functions identified using the ssGSEA algorithm. The heatmap was generated using R software (version 4.1.1, https://www.r-project.org/). Adjustments to font type and size were made for enhanced clarity. (**b**) Correlation matrix displays the proportions of different immune cell types. (**c**) Correlation matrix presents the proportions of various immune functions. (**d**) Immune Cell Profile in AA and Normal Scalp Tissue: The box plot compares 16 immune cell (imc) populations between Alopecia Areata-affected scalp (AA, red boxes) and normal scalp from healthy controls and unaffected AA scalp (Normal, green boxes). Significant increases in imc subsets, notably CD8 + T cells, are observed in AA lesions, except for T follicular helper (Tfh) and regulatory T cells (Treg). Significance is marked by *p < 0.05, **p < 0.01, ***p < 0.001, with 'ns' for non-significant. The box encompasses the interquartile range with the median indicated by the horizontal line, and outliers as points. (e) Immune Function Enhancement in AA Scalp: The box plot shows a marked elevation of 13 immune function (imf) parameters in AA compared to normal scalp tissue. Enhancements in cytolytic activity and inflammation promotion are the most pronounced, highlighting their potential role in AA pathophysiology. Statistical significance is denoted by *p < 0.05, **p < 0.01, ***p < 0.001. The plot's box represents the interquartile range, the median is the line within, and points are outliers. AA, alopecia areata, imc, immune cell; imf, immune function; ssGSEA, single-sample gene set enrichment analysis, Tfh, T follicular helper cells; Treg, regulatory T cells.

We applied the ssGSEA algorithm for a comparative analysis of immune cell infiltration in AA lesions versus NC scalp. The analysis incorporated 16 types of imc and 13 types of imf across 60 AA samples, as shown in Fig. 3a. Correlations between imc and imf proportions were further explored in Fig. 3b,c. Significantly, Fig. 3d,e reveal a pronounced increase in various imc and imf in AA compared to NC. Figure 3d illustrates marked increments in specific imc subsets, particularly CD8 + T cells, in AA lesions, excluding T follicular helper (Tfh) and regulatory T cells (Treg). Figure 3e highlights enhanced immune functions in AA, with notable upregulation in cytolytic activity and inflammation promotion. These findings underscore the extensive inflammatory infiltration characterizing AA.

### Analysis of FRDEGs combined with expression immune cell infiltration

**Figure 4:**
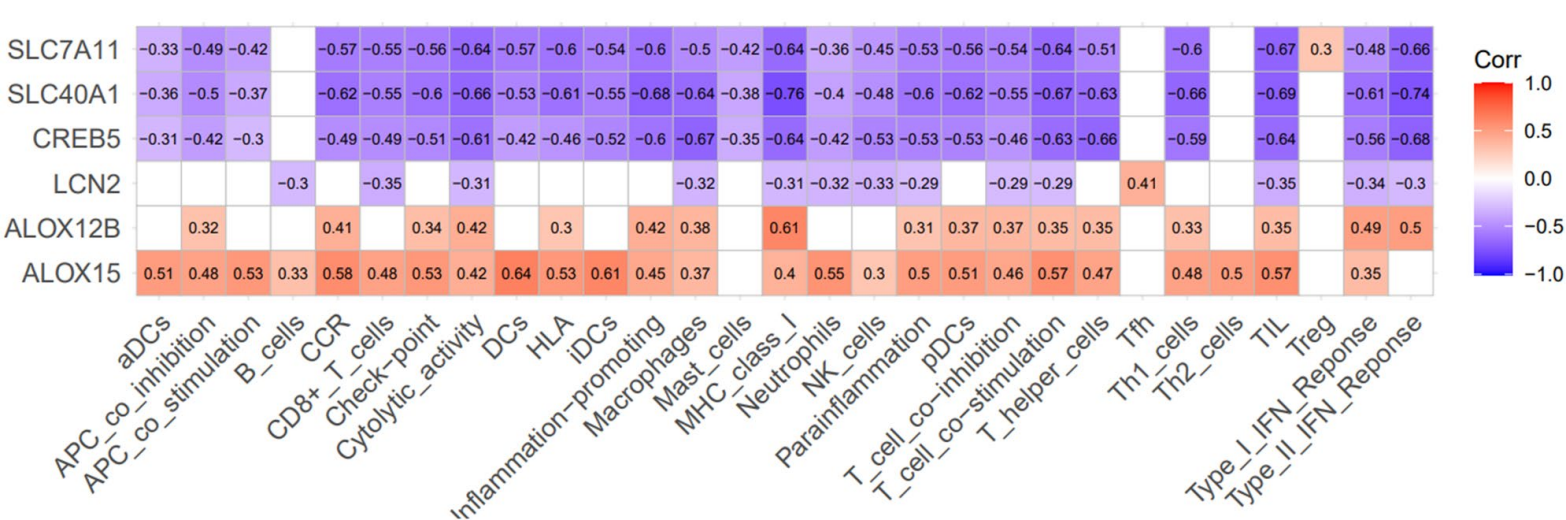
Correlation Analysis of FRDEGs with Immune Cells (imc) and Immune Functions (imf) in Alopecia Areata (AA). This figure illustrates the correlation between ferroptosis-related differentially expressed genes (FRDEGs) and immune cell infiltration. Notably, ALOX12B and ALOX15 demonstrated a positive correlation with both imc and imf. In contrast, SLC40A1, LCN2, CREB5, and SLC7A11 showed a predominantly negative correlation with these parameters. AA, alopecia areata; imc, immune cells; imf, immune functions.

For correlation analysis, we assembled data from six FRDEGs, 16 imcs, and 13 imfs associated with immune cell infiltration and generated a corresponding heatmap. A strong correlation between these genes and immune cell infiltration was observed. Among them, ALOX12B and ALOX15 were positively correlated with imc and imf, while SLC40A1, LCN2, CREB5, and SLC7A11 were predominantly negatively correlated (Fig. 4).

### Marker gene identification via machine learning

**Figure 5:**
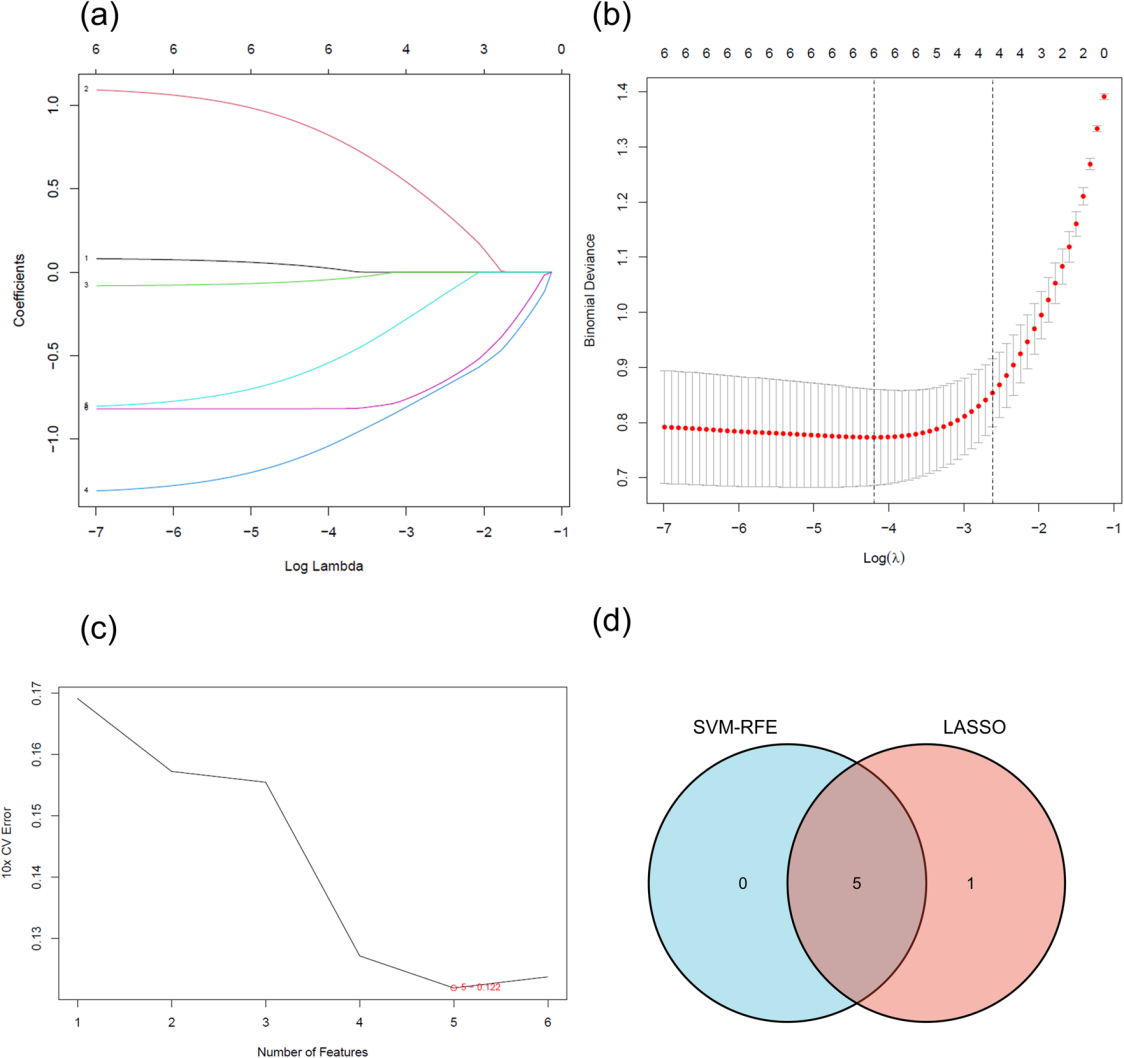
Diagnostic Marker Gene Identification for Alopecia Areata (AA). (**a**) Displays ten-fold cross-validation in the LASSO model, with each curve representing an individual gene. (**b**) Shows feature selection in the LASSO model, highlighting optimal lambda values with vertical dashed lines. (**c**) Depicts the process of AA marker gene identification using the SVM-RFE algorithm. (**d**) Venn diagram demonstrates the overlap of genes identified by both SVM-RFE and LASSO methods. Five overlapping genes—ALOX15, SLC40A1, LCN2, CREB5, and SLC7A11—were identified as potential diagnostic markers for AA. AA, alopecia areata, SVM-RFE, support vector machine-recursive feature elimination; LASSO, least absolute shrinkage and selection operator;)

We utilized two distinct algorithms to identify potential marker genes. The LASSO regression algorithm identified six FEDEGs as AA markers (Fig. 5a,b). The SVM-RFE algorithm determined five genes in FEDEGs (Fig. 5c). Ultimately, five overlapping genes (ALOX15, SLC40A1, LCN2, CREB5, and SLC7A11) were selected (Fig. 5d), potentially representing key genes in AA progression.

### Hierarchical and external validation of marker genes

**Figure 6:**
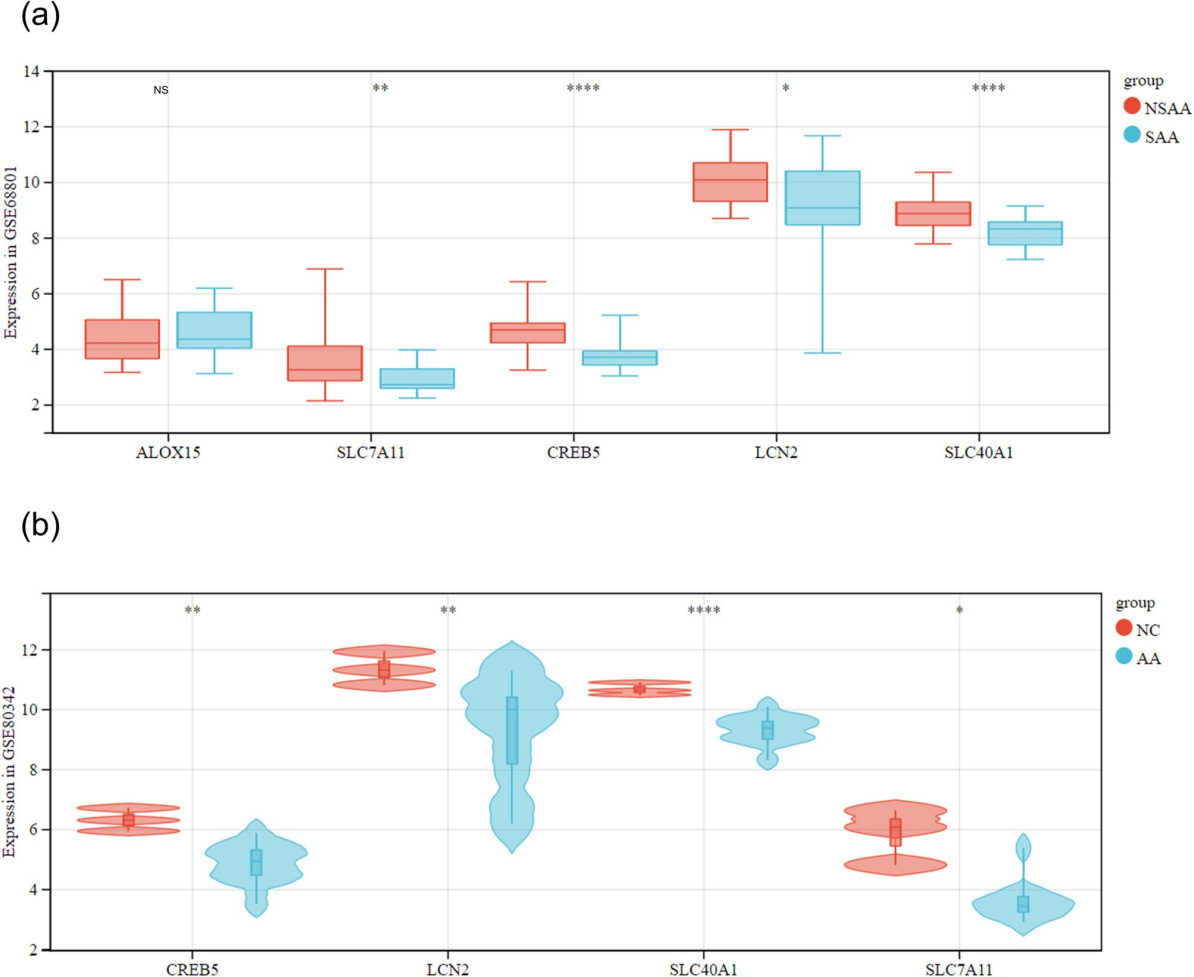
Validation of Marker Genes for Alopecia Areata (AA). (**a**) Hierarchical validation in the GSE68801 dataset, with subgroups SAA (32 cases) and NSAA (28 cases) based on clinical parameters. SLC40A1, LCN2, CREB5, and SLC7A11 showed significantly lower expression in the SAA group. (**b**) External validation using the GSE80342 dataset (12 AA lesions, 3 NC scalps) revealed significant differences in gene expression between AA and NC groups, particularly higher expression of SLC40A1, LCN2, CREB5, and SLC7A11 in AA. AA, alopecia areata patients; NC, normal controls.

For assessing the diagnostic efficacy of the five marker genes, the AA samples from GSE68801 were categorized into two subgroups (SAA and NSAA) based on clinical parameters. The SAA group contained 32 cases, and the NSAA group had 28 cases. Only SLC40A1, LCN2, CREB5, and SLC7A11 showed significantly lower expression in the SAA group, while ALOX15 exhibited no significant difference between groups (Fig. 6a). To further validate the marker genes' diagnostic efficacy, we extracted gene expression data from GSE80342 (12 AA lesions and 3 NC scalps) and generated a violin plot (Fig. 6b). The expression of these four genes was significantly different between AA and NC groups, with SLC40A1, LCN2, CREB5, and SLC7A11 displaying significantly higher expression in AA, consistent with GSE68801 datasets.

### Development of a nomogram for predicting AA risk.

**Figure 7:**
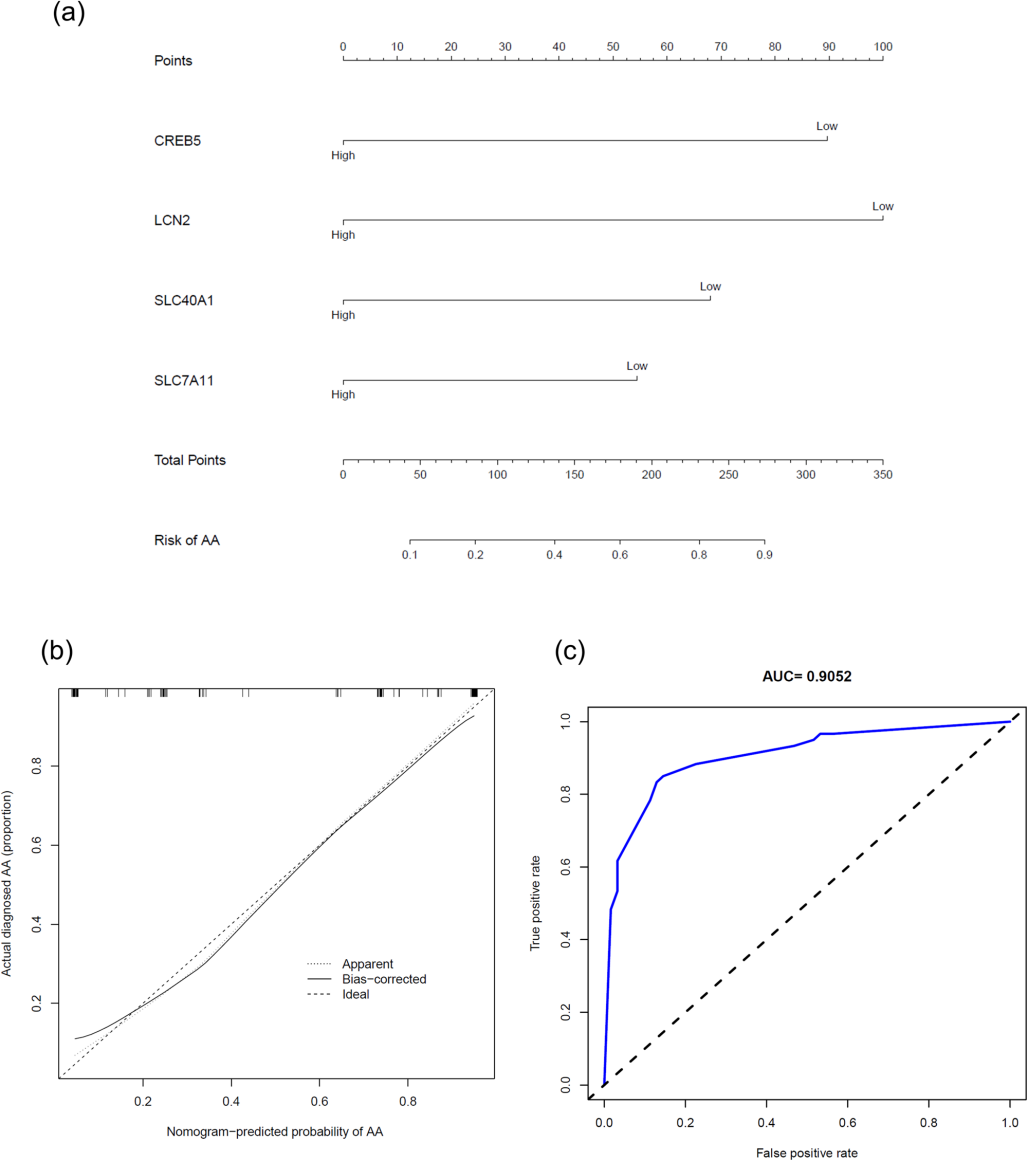
Nomogram for Predicting the Risk of Alopecia Areata (AA). (**a**) A nomogram developed using the GSE68801 dataset, incorporating selected marker genes for AA diagnosis. Each gene contributes a score, with the total score indicating the AA risk level. (**b**) Calibration curve assessing the nomogram's accuracy, where a line closer to the ideal line represents higher predictive accuracy for AA. (**c**) Receiver Operating Characteristic (ROC) curve for the AA diagnosis using marker genes from the GSE68801 dataset, exhibiting an area under the curve (AUC) of 0.9052, indicating high diagnostic performance. AA, alopecia areata; ROC, receiver operating characteristic; AUC, area under the curve.

To determine the predictive potential of the three identified marker genes for AA risk, we established a multivariate logistic model (Fig. 7a). In the nomogram, each marker gene was assigned a score, and the total score was calculated by summing the individual scores. The total score correlated with varying AA risks. The calibration curve (Fig. 7b) demonstrated the nomogram's accuracy; a bias-corrected line closer to the ideal line indicated higher prediction accuracy for AA. Our model exhibited high accuracy. To highlight the predictive capacity of these four marker genes, we integrated them into a receiver operating characteristic (ROC) curve (Fig. 7c), which yielded an area under the curve (AUC) of 0.9052. This value signified that the four marker genes provided strong predictive performance for AA.

### Immunofluorescence

**Figure 8:**
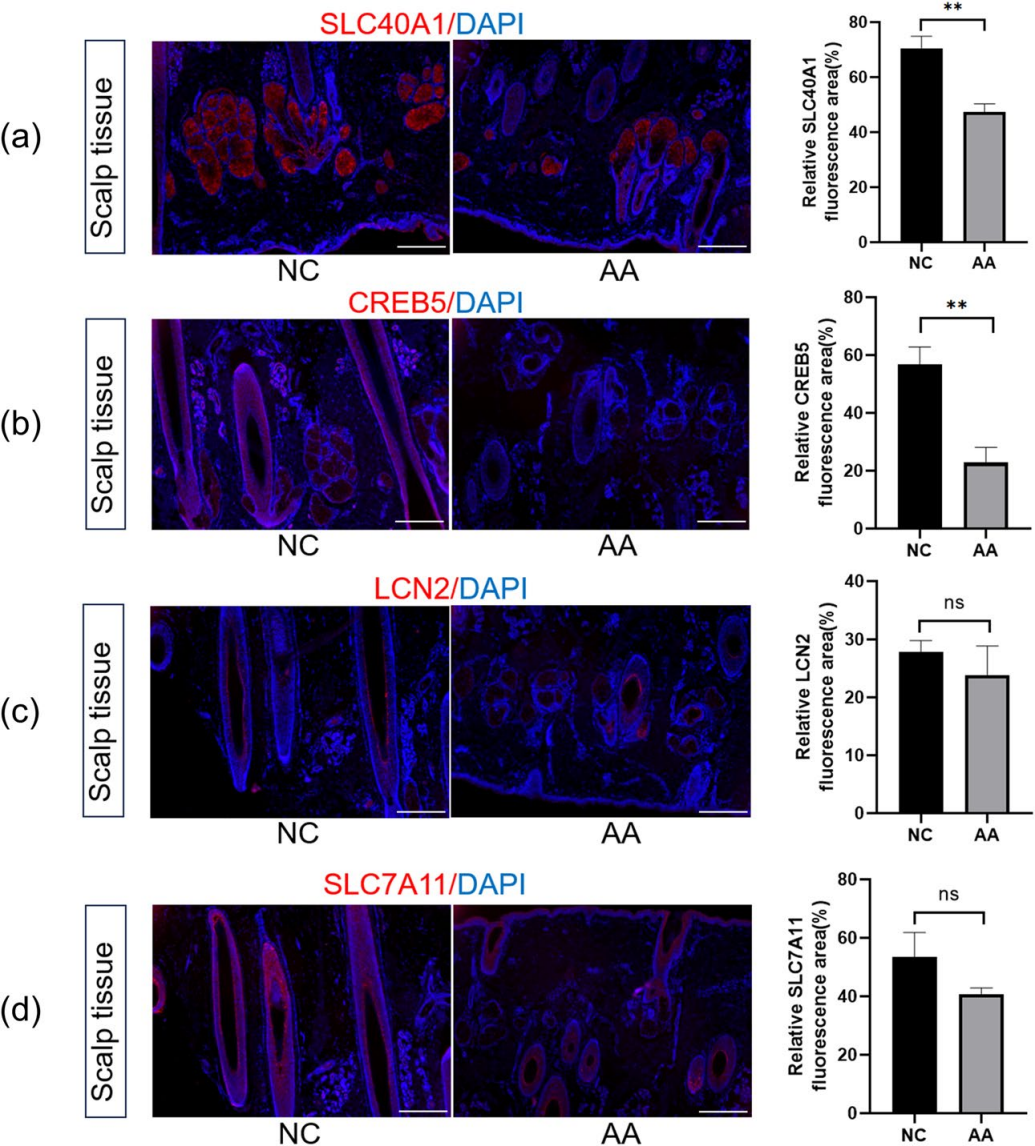
Differential Expression of SLC40A1, CREB5, LCN2, and SLC7A11 in Scalp Tissues of Alopecia Areata (AA) Patients and Normal Controls (NC). (**a**–**d**) Fluorescent micrographs depict the staining intensity of SLC40A1, CREB5, LCN2, and SLC7A11 (red), with nuclei counterstained with DAPI (blue). Quantitative analysis demonstrates a notable downregulation of SLC40A1 and CREB5 in the scalp tissues of AA patients relative to NC, as reflected by the diminished fluorescence intensity. In contrast, LCN2 and SLC7A11 show no significant change. Data are expressed as the mean ± S.E.M. Asterisks indicate statistical significance where **P < 0.01; 'ns' denotes non-significance. Scale bar represents 500 μm. AA, alopecia areata patients; NC, normal controls; DAPI, 4',6-diamidino-2-phenylindole.

We evaluated the expression of the respective genes in scalp tissues from 3 AA patients and 3 NC. As depicted in (Fig. 8), immunofluorescence staining confirmed reduced expression of SLC40A1, CREB5, LCN2, and SLC7A11 in the scalp tissues of AA patients compared to healthy controls, with SLC40A1 and CREB5 showing statistically significant differences. Furthermore, LCN2, CREB5, and SLC7A11 were predominantly localized to the outer root sheath, whereas SLC40A1 was primarily observed in the sebaceous gland region.

## Discussion

In our investigation, utilizing advanced machine learning methodologies, we pinpointed SLC40A1, LCN2, CREB5, and SLC7A11 as prospective diagnostic biomarkers for AA. Distinctly, our findings bridge, for the first time, the association between ferroptosis and the pathogenesis of AA, laying foundational groundwork for further exploration into the role of ferroptosis in this condition.

AA affects approximately 2% of the global population^8^, with profound impacts on quality of life and potential psychological ramifications^26^. Its links to cardiovascular disease underline the necessity for early diagnosis. Despite some molecular indications suggesting ferroptosis as a pathophysiological factor, the connection remained largely speculative until our study.

Excess iron is fundamental to the process of ferroptosis. Fe2 + catalyzes the formation of reactive oxygen species (ROS) through the Fenton reaction, leading to lipid peroxidation and the initiation of ferroptosis^27^. Iron contributes to the generation of highly reactive and toxic hydroxyl radicals, thereby stimulating oxidative damage. Recent literature suggests that aberrations in iron metabolism may play a crucial role in skin aging^28^. Studies employing iron chelators to prevent ROS generation have shown promise in reducing photodamage and aging-related oxidative stress^29^. Research on ferroptosis in skin diseases has been limited, mostly focusing on autoimmune disorders such as systemic lupus erythematosus (SLE) and vitiligo. In these conditions, oxidative stress plays a dominant role, leading to the ferroptosis of neutrophils and melanocytes^30^. However, research on the role of ferroptosis in AA, particularly its specific pathological contributions and potential therapeutic implications, remains relatively scarce.

Recent studies hint at a link between ferroptosis and immune cell activity, exemplified by the role of CD8 + T cells in intensifying ferroptosis in tumor cells^31^. This connection is particularly pertinent to AA, where both ferroptosis and immune dysfunction appear intertwined. Our analysis delves into this interplay, revealing new aspects of AA’s immunopathology. Our data align with recent single-cell sequencing results^32^, indicating that CD8 + T cells, marked by heightened cytolytic activity and inflammation, are the significant drivers of the condition.

While previous studies primarily utilized bioinformatics to discern potential AA marker genes—such as Yuan X et al. pinpointing EOMEs as a potential therapeutic target for AA^33^, Zhang T et al. highlighting four key genes (CD28, HOXC13, KRTAP1-3, and GPRC5D) for AA treatment^34^, and Zhang Z et al. identifying three salient genes (BMP2, KRTs, and KRTAPs) in relation to AA^35^—our research stands distinct.

By synergistically integrating the GEO dataset, the FerrDb database, and advanced machine learning techniques, we have delineated five potential marker genes specifically associated with both ferroptosis and AA, underscoring a novel dimension to the molecular understanding of the disease. Notably, four of these genes (SLC40A1, LCN2, CREB5, and SLC7A11) were found to be downregulated in AA lesions compared to NC scalps. Their expression levels inversely correlated with AA severity. These findings are further supported by the high accuracy of our ROC curves, suggesting their potential as AA biomarkers.

Recent studies have highlighted the role of SLC40A1, a protein localized to the cellular membrane, with a putative function in iron export from duodenal epithelial cells^36^. This discovery broadens our understanding of iron metabolism, a key factor in ferroptosis. Another gene, LCN2, is known for encoding a protein that is primarily involved in lipid transport and innate immunity. It may also play a role in maintaining skin homeostasis and defending against pathogenic invasion^37^. Interestingly, therapies targeting LCN2 have been shown to induce ferroptosis in hepatic tumor cells, offering new avenues for hepatocarcinoma treatment^37^. The CREB5 gene is implicated in the PI3K-Akt signaling pathway^38^. Its protein product, predominantly expressed in articular cartilage, acts as a transcriptional factor to enhance Prg4 expression. This activation is crucial for the TGF-β and EGFR pathways, both important in hair follicle morphogenesis^39^. The role of CREB5 in the pathogenesis of AA, however, remains to be elucidated. Additionally, SLC7A11, a relative of SLC40A1, is involved in iron efflux and exosome-mediated ferritin output, which may inhibit ferroptosis. Its role as a potential marker in ferroptosis pathways, specifically in the context of AA, merits further investigation^40^. While research on SLC7A11 is predominantly focused on oncology, its relevance to AA is an emerging area of interest, especially considering its function in amino acid transport, which may prevent lipid peroxidation and ferroptosis^41^.

Immunofluorescence demonstrated diminished expression of SLC40A1, CREB5, LCN2, and SLC7A11 in the scalp tissues of AA patients compared to NC, with SLC40A1 and CREB5 showing statistically significant differences. The absence of statistical differences for some molecules might be attributed to the limited number of cases selected for immunofluorescence. Nevertheless, a decline in the expression of these molecules in AA was evident. Notably, these molecules were primarily localized in the outer root sheath and sebaceous gland regions, areas known for their metabolic activity. This suggests a potential link between AA onset and metabolic perturbations.

While our research makes important connections between ferroptosis and AA, there are limitations to consider. The small size of our validation dataset could affect the reliability of our conclusions. Specifically, the observed lower levels of LCN2 and SLC7A11 in AA, given the limited statistical power of our study, may not fully capture their role in the disease. These results, therefore, should be viewed as indicative rather than definitive, pending further investigation with larger cohorts and functional validation.

In conclusion, our findings elucidate the potential role of FRGs in AA's progression. This not only enriches our understanding of AA's molecular underpinnings but also points toward promising diagnostic and therapeutic avenues. Our results beckon more exhaustive studies, aiming for personalized treatment solutions for AA patients.

### Supplementary Information

Supplementary Information.

## Data Availability

The data used to support the findings of the research are available from the corresponding author upon request.

## Abbreviations

AA: Alopecia areata
FRDEGs: Ferroptosis-related differentially expressed genes
ssGSEA: Single-sample gene set enrichment analysis
SVM-RFE: Support vector machine-recursive feature elimination
LASSO: Least absolute shrinkage and selection operator
FRGs: Ferroptosis-related genes
PAA: Patchy alopecia areata
AT: Alopecia totalis
AU: Alopecia universalis
SAA: Severe alopecia areata
NSAA: Considered non-severe alopecia areata
FRDEGs: Ferroptosis-related differentially expressed genes
DEGs: Differentially expressed genes
imc: Immune cells
imf: Immune functions
SVM: Support vector machine
RFE: Recursive feature elimination
ROC: Receiver operating characteristic
AUC: Area under the curve
NC: Normal control
DAPI: 4',6-Diamidino-2-phenylindole
FDR: False Discovery Rate
ROS: Reactive oxygen species
SLE: Systemic lupus erythematosus
Tfh: T follicular helper cells
Treg: Regulatory T cells

## Code availability

The codes and algorithms used in this study are currently not publicly available due to ongoing research and intellectual property considerations. However, we are willing to share them with other researchers for non-commercial academic use, subject to specific requests and a confidentiality agreement. Interested parties should contact the corresponding author for further information.
